# Epigenetic Profiles of Triple-Negative Breast Cancers of African American and White Females

**DOI:** 10.1001/jamanetworkopen.2023.35821

**Published:** 2023-10-05

**Authors:** Miquel Ensenyat-Mendez, Maria Solivellas-Pieras, Pere Llinàs-Arias, Sandra Íñiguez-Muñoz, Jennifer L. Baker, Diego M. Marzese, Maggie L. DiNome

**Affiliations:** 1 Cancer Epigenetics Laboratory at the Cancer Cell Biology Group, Health Research Institute of the Balearic Islands, Palma, Spain; 2 Department of Surgery, David Geffen School of Medicine, University California, Los Angeles; 3 Department of Surgery, Duke University School of Medicine, Durham, North Carolina

## Abstract

**Importance:**

Triple-negative breast cancer (TNBC) is the most aggressive breast cancer subtype and appears to have disproportionately higher incidence and worse outcomes among younger African American females.

**Objective:**

To investigate whether epigenetic differences exist in TNBCs of younger African American females that may explain clinical disparities seen in this patient group.

**Design, Setting, and Participants:**

This cross-sectional study used clinical, demographic, DNA methylation (HumanMethylation450; Illumina), and gene expression (RNA sequencing) data for US patient populations from publicly available data repositories (The Cancer Genome Atlas [TCGA], 2006-2012, and Gene Expression Omnibus [GEO], 2004-2013) accessed on April 13, 2021. White and African American females with TNBC identified in TCGA (69 patients) and a validation cohort of 210 African American patients from GEO (GSE142102) were included. Patients without available race or age data were excluded. Data were analyzed from September 2022 through April 2023.

**Main Outcomes and Measures:**

DNA methylation and gene expression profiles of TNBC tumors by race (self-reported) and age were assessed. Age was considered a dichotomous variable using age 50 years as the cutoff (younger [<50 years] vs older [≥50 years]).

**Results:**

A total of 69 female patients (34 African American [49.3%] and 35 White [50.7%]; mean [SD; range] age, 55.7 [11.6; 29-82] years) with TNBC were included in the DNA methylation analysis; these patients and 210 patients in the validation cohort were included in the gene expression analysis (279 patients). There were 1115 differentially methylated sites among younger African American females. The DNA methylation landscape on TNBC tumors in this population had increased odds of enrichment of hormone (odds ratio [OR], 1.82; 95% CI, 1.21 to 2.67; *P* = .003), muscle (OR, 1.85; 95% CI, 1.44 to 2.36; *P* < .001), and proliferation (OR, 3.14; 95% CI, 2.71 to 3.64; *P* < .001) pathways vs other groups (older African American females and all White females). Alterations in regulators of these molecular features in TNBCs of younger African American females were identified involving hormone modulation (downregulation of androgen receptor: fold change [FC] = −2.93; 95% CI, −4.76 to −2.11; *P* < .001) and upregulation of estrogen-related receptor α (FC = 0.86; 95% CI, 0.34 to 1.38; *P* = .002), muscle metabolism (upregulation of *FOXC1*: FC = 1.33; 95% CI, 0.62 to 2.03; *P* < .001), and proliferation mediators (upregulation of *NOTCH1*: FC = 0.71; 95% CI, 0.23 to 1.19; *P* = .004 and *MYC* (FC = 0.81; 95% CI, 0.18 to 1.45; *P* = .01).

**Conclusions and Relevance:**

These findings suggest that TNBC of younger African American females may represent a distinct epigenetic entity and offer novel insight into molecular alterations associated with TNBCs of this population. Understanding these epigenetic differences may lead to the development of more effective therapies for younger African American females, who have the highest incidence and worst outcomes from TNBC of any patient group.

## Key Points

**Question:**

Do epigenetic differences exist in triple-negative breast cancer (TNBC) tumors of younger African American patients compared with White and older African American patients?

**Findings:**

In this cross-sectional study of 69 female patients with TNBC, tumors of younger African American patients had a distinct DNA methylation profile compared with those of older African American females and White females of all ages. This unique epigenetic landscape involved hormone, muscle, and proliferation pathways, which may be associated with the increased aggressiveness and more basal phenotype in tumors of this patient group.

**Meaning:**

These findings suggest that TNBC tumors of younger African American females may be a distinct entity clinically and epigenetically.

## Teaser

This cross-sectional study investigates epigenetic differences in triple-negative breast cancer among younger African American females vs other age and race groups.

## Introduction

Breast cancer is a heterogeneous disease divided into 4 main subtypes based on distinct pathological and molecular features. Triple-negative breast cancer (TNBC), which lacks estrogen receptor (ER) and progesterone receptor expression and human epidermal growth factor receptor 2 (*ERBB2*, formerly *HER2*) overexpression, is the most aggressive subtype, with more patients progressing to metastatic disease than any other breast cancer subtype. African American females develop TNBC at more than twice the rate of White females and have a one-third higher risk of dying from this disease.^1,2,3^ Younger African American females have the highest incidence of TNBC (39%) compared with older African American females (14%) and White females of all ages (16%).^4^ The basis of this marked disparity in incidence and outcome is poorly understood.

Studies have suggested that socioeconomic factors and treatment variables may account for the disparities in survival.^5,6,7^ However, studies from 2014^2^ and 2009^3^ suggested that biological differences may also contribute. We and others found that after adjusting for socioeconomic, treatment, and tumor variables, younger African American females with TNBC had lower rates of response to chemotherapy than younger White females.^8,9^ Furthermore, African American females had a higher proportion of basal-like and mesenchymal stem–like tumors, subtypes of TNBC associated with worse prognoses.^10^ Understanding molecular differences that contribute to the more aggressive behavior of TNBC in this patient population is essential for improving outcomes.

Epigenetic modifications, such as DNA methylation (DNAm), represent changes to the structure of DNA that affect gene expression without altering the underlying gene sequence. The epigenetic landscape is determined by a person’s genetic makeup and a variety of environmental factors, such as diet, stress, and toxins. Importantly, epigenetic alterations have been implicated in breast cancer development and progression. A 2012 study^11^ suggested that TNBC was epigenetically distinct from other breast cancer subtypes, and we previously identified 4 prognostically relevant epigenetic subtypes within TNBC.^12^

However, the contribution of epigenetics to the pathogenesis of TNBC by race and age remains poorly understood. In this exploratory study, we sought to identify whether TNBCs in younger African American females have epigenetic differences associated with altered molecular pathways that may contribute to the higher incidence and lower survival seen in this patient group.

## Methods

Data for this cross-sectional study were gathered from publicly available data sets and did not require institutional review board approval or patient informed consent per the Code of Federal Regulations (45 CFR §46.104). This study followed the Strengthening the Reporting of Observational Studies in Epidemiology (STROBE) reporting guideline.

### Data Collection and Normalization

**Figure 1.**
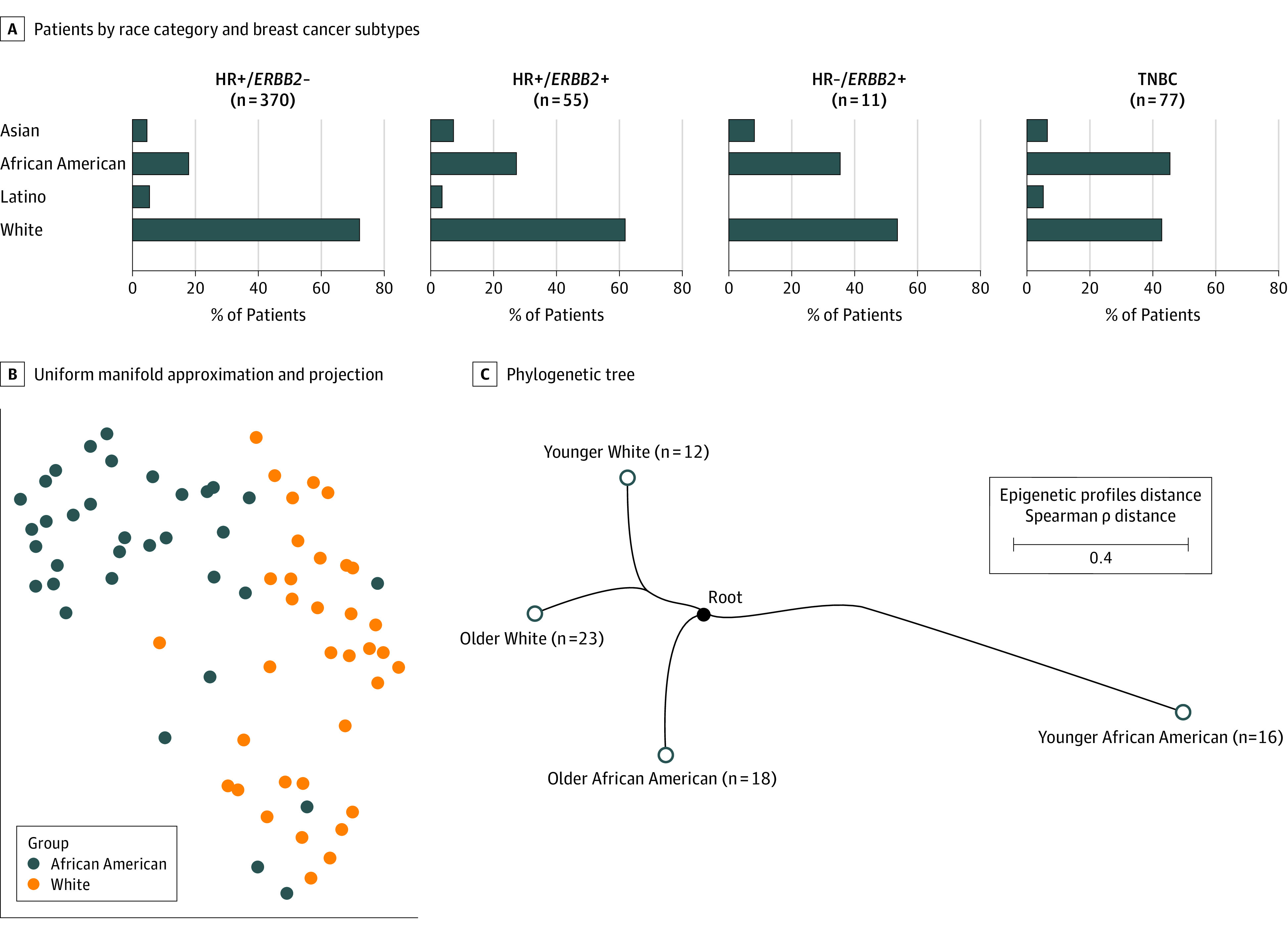
DNA Methylation Differences in Breast Cancer by Race A, The percentage of patients by race category among breast cancer subtypes is presented. B, The uniform manifold approximation and projection displays the distribution of triple-negative breast cancer (TNBC) specimens according to differentially methylated sites. C, The phylogenetic tree representing the DNA methylation–based Spearman ρ distance between TNBC tumors from different race and age categories. *ERBB2* indicates human epidermal growth factor receptor 2; HR, hormone receptor.

Clinical and demographic data from US patients with TNBC in the Cancer Genome Atlas (TCGA) 2006 to 2012 were obtained from the National Cancer Institute Genomic Data Commons portal. Race (African American, American Indian or Alaska Native, Asian, or White) and ethnicity (Hispanic or non-Hispanic) for each participant was self-reported and annotated by TCGA data abstractors. Ethnicity was considered to compare the incidence of TNBC by race and ethnicity group, but ethnicity was not used as a classification variable for the remainder of the study. Groups were generated based on race and age. Only treatment-naive tumors from African American and White patients were included in the study given the small number of patients of other races with available DNAm and gene expression data. DNAm (HumanMethylation450 array; Illumina) and gene expression (RNA sequencing) data were downloaded using the R statistical software package TCGAbiolinks version 2.16.4 on April 13, 2021. Patients of other races or without race or age data were excluded (88 patients). Groups represented in Figure 1 include non-Hispanic African American, non-Hispanic Asian, Hispanic or Latino White, and non-Hispanic White patients. DNA methylation and gene expression data were from patients self-reported as African American or White based on race alone, and ethnicity was not included (given the small number of Hispanic patients with DNAm data). African American patients with TNBC who had RNA sequencing data enrolled in the Gene Expression Omnibus (GEO; GSE142102) 2004 to 2013 were included as a validation cohort. Gene expression data were normalized to *Z* score using the scale command in R. Probes not passing GenomeStudio quality controls (Illumina) and those with known cross-reactivity or missing data were excluded. All probes with single nucleotide variations (formerly single nucleotide polymorphisms) located in or near (within 25 base pairs) repetitive elements were removed. DNAm levels were obtained as β values. Chromatin immunoprecipitation sequencing of estrogen-related receptor α (*ESRRA*) binding sites were downloaded from the University of California, Santa Cruz Genome Browser and androgen receptor (AR) binding sites from GEO (GSE83860).

### Statistical Analysis

Patients were grouped based on race and age into 4 groups: younger African American (aged <50 years), older African American (aged ≥50 years), younger White (aged <50 years), and older White (aged ≥50 years) female patients. Age was dichotomized based on studies^9^ finding an increased risk of mortality in African American patients younger than 50 years. The Uniform Manifold Approximation and Projection for Dimension Reduction (UMAP) technique was used to visualize the distribution of groups using the R M3C package version 1.18.0. Heat maps were used to visualize hierarchical cluster analyses based on Euclidean distance using the R package gplots version 3.1.3. Correlation matrices were computed using R statistical software version 4.2.0 (R Project for Statistical Computing) and plotted using the R gplots2 package. Phylogenetic trees with Spearman ρ distances between DNAm profiles of the 4 groups were generated using the FigTree software version 1.4.3 (Andrew Rambaut Lab, University of Edinburgh). We compared categorical variables using the Fisher exact test and age with the Kruskal-Wallis test. Differences between groups were computed using Dunn multiple comparison tests in R. Pathway enrichment scores were estimated using the hypergeometric test, and the χ^2^ test was used to calculate the *P* value. A 2-sided *P* value < .05 was considered significant. Gene set enrichment analysis (GSEA) was performed using GSEA software version 4.3.2 (University of California, San Diego, and the Broad Institute) downloaded from the project website.^13,14,15^ Pathway visualization was performed using Cytoscape and the KEGGscape plugin.^16,17^ Data were analyzed from September 2022 through April 2023.

Differentially methylated sites (DMSs) were computed using the Wilcoxon test. All CpG sites with a differential mean β value of 15% or greater and a *P* value < .05 were considered DMSs. For the gene expression analysis, the number of counts was converted to a log2 value. The parametric *t* test was used to assess the significance of differentially expressed genes (DEGs), defined as an absolute fold difference between groups greater than 0.7 and a *P* value < .05. Expression-methylation quantitative trait loci (emQTL) involving 9398 CpGs in 870 genes were obtained from Fleischer et al.^18^ The Genomic Regions Enrichment of Annotations Tool (Stanford University) was used to identify genes with DMSs. Pathway enrichment analysis was performed using the R GOfuncR package version 1.16.0. Genes associated with relevant pathways were selected using the R biomaRT package version 2.52.0. Pathway enrichment was represented using the R ggplot2 package version 3.3.6.

## Results

### Patient and Clinicopathologic Data

We identified 69 female patients (34 African American [49.3%] and 35 White [50.7%]; mean [SD; range] age, 55.7 [11.6; 29-82] years), including 16 younger African American (mean [SD; range] age, 45.8 [4.1; 35-50] years), 18 older African American (mean [SD; range] age, 64.9 [9.4; 51-80] years), 12 younger White (mean [SD; range] age, 44.2 [6.4; 29-50] years), and 23 older White (mean [SD; range] age, 61.4 [8.3; 51-82] years) females. African American patients had proportionally higher rates of TNBC than patients with other races and ethnicities (Figure 1A). For this analysis, self-reported Latino or Hispanic females were considered as a group to establish the proportion of patients with TNBC. This population included 4 Hispanic or Latino White (5.2%), 35 non-Hispanic African American (45.5%), 5 non-Hispanic Asian (6.5%), and 33 non-Hispanic White (42.8%) females. However, due to the small proportion in this group, ethnicity was removed as a classification factor for further analyses. When including only patients with DNAm data, our study population included 35 White individuals (2 Hispanic White and 33 non-Hispanic White individuals) and 34 non-Hispanic African American individuals. Tumor histology, location, stage, and nodal status were similar between groups (eTable in Supplement 1).

### Epigenetic Differences by Race

We identified 164 DMSs between African American and White patients with TNBC (eFigure 1A in Supplement 1), which demonstrated a modest ability to distinguish patients based on race (Figure 1B; eFigure 1B in Supplement 1). Therefore, we added age to further stratify groups. By comparing DNAm levels of the top 5000 most variable genomic regions, we found that TNBCs of younger African American patients displayed a distinct epigenetic profile compared with TNBCs of other groups (younger White , older White, and older African American patients) (eFigure 1C in Supplement 1). This observation persisted when we examined the top 2000 and top 1000 most variable regions (eFigure 1D-E in Supplement 1). However, when we selected less than 1000 regions, TNBCs of younger African American females clustered with those of older African American females and remained separate from White females of any age (eFigure 1F-G in Supplement 1). This suggests that this small set of hypervariable genomic regions (<500 regions) was associated more with race than age. TNBCs of younger African American patients displayed a longer statistical distance from other patient groups (Figure 1C), indicating a distinct epigenetic landscape.

### Association of Epigenetic Alterations With Pathways in Younger African American Patients

**Figure 2.**
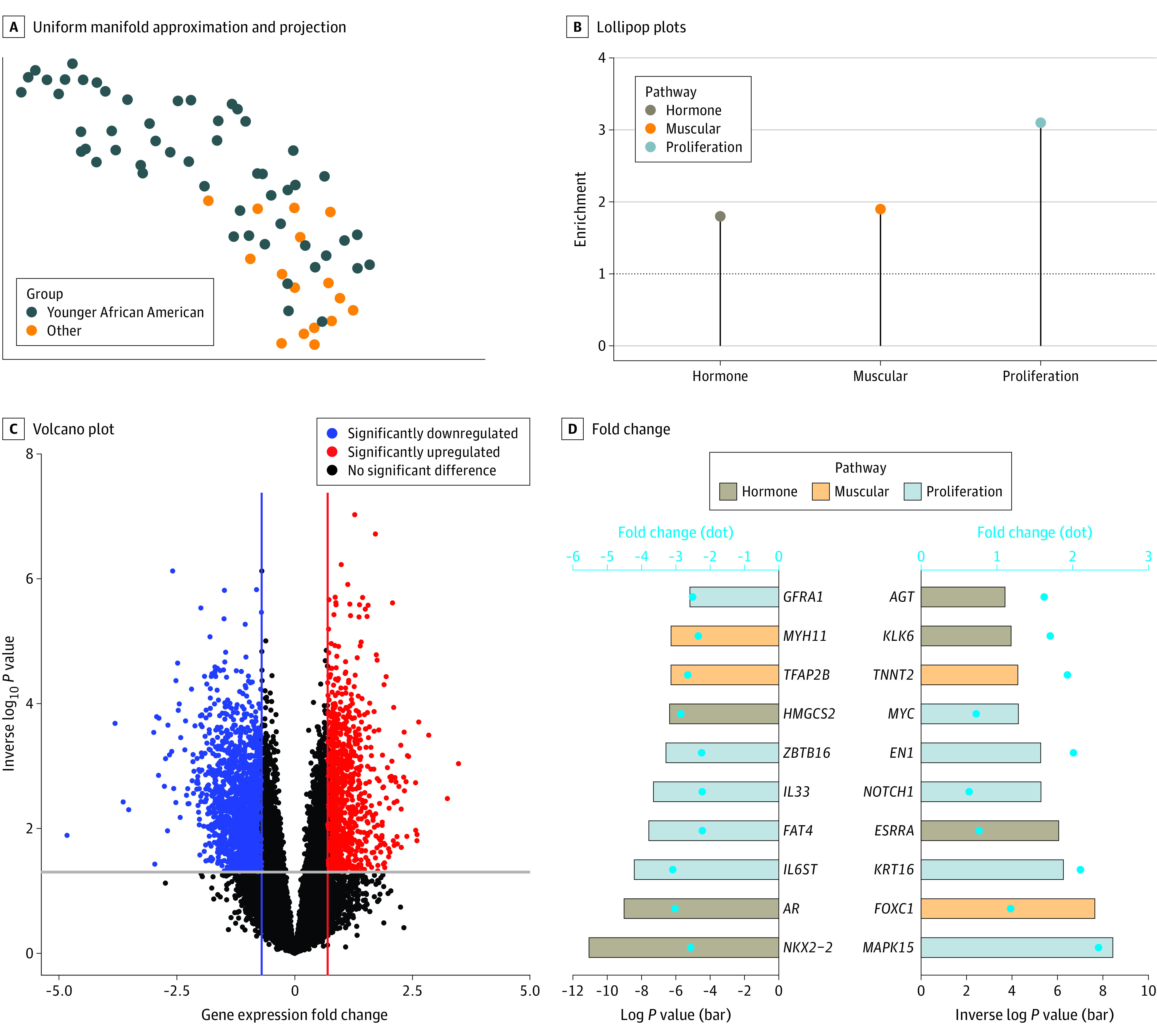
DNA Methylation, Gene Expression, and Pathway Enrichment in Younger African American Compared With White and Older African American Patients A, The uniform manifold approximation and projection displays the distribution of triple-negative breast cancer specimens according to differentially methylated sites between younger (aged <50 years) African American females and other groups (older [aged ≥50 years] African American, younger White, and older White females). B, Lollipop plots represent enrichment values for hormone, muscle, and proliferation pathways. C, The volcano plot represents the gene expression fold change and −log10 of the *P* value between triple-negative breast cancer from younger African American females and other groups. D, The bar plot shows the fold change (dots) and −log10 *P* value (bars) of genes associated with hormone, muscle, and proliferation pathways. AR indicates androgen receptor.

Among 1115 DMSs between younger African American patients and other groups, we identified 80 hypermethylated and 1035 hypomethylated regions (eFigure 2A in Supplement 1). In hierarchical clustering and UMAP analyses, these regions effectively separated younger African American patients from other groups (Figure 2A; eFigure 2B in Supplement 1). We conducted associative pathways enrichment analyses using genes located near the younger African American patient–specific hypomethylated sites and found significantly increased odds of enrichment in hormone (odds ratio [OR], 1.82; 95% CI, 1.21-2.67; *P* = .003), muscle (OR, 1.85; 95% CI, 1.44-2.36; *P* < .001), and proliferation (OR, 3.14; 95% CI, 2.71-3.64; *P* < .001) pathways compared with other groups (older African American females and White females of all ages) (Figure 2B).

We integrated gene expression profiles and identified 2864 DEGs, of which 1073 DEGs were upregulated and 1791 DEGs were downregulated in TNBCs of younger African American patients (Figure 2C). A significant proportion of these DEGs had emQTL (OR, 1.29; 95% CI, 1.18 to 1.40; *P* < .001), underscoring the importance of epigenetic regulation in maintaining the specific transcriptome of TNBCs of younger African American patients (eFigure 2C in Supplement 1). Similar to epigenetic alterations, gene expression changes were identified in hormone, muscle, and proliferation pathways. Downregulation of AR and upregulation of *ESRRA* were among the most relevant DEGs involved in hormone metabolism. We additionally identified significant upregulation of *FOXC1*, a transcription factor involved in muscle cell metabolism, and upregulation of *MYC* and *NOTCH1*, 2 key proliferative mediators (Figure 2D). The gene set enrichment analysis using gene expression data revealed a decreased response to hormones, specifically estradiol, in younger African American patients and an activation of gene networks involved in cell cycle proliferation (eFigure 2D-E in Supplement 1).

### Epigenetic Differences by Age in African American Patients

**Figure 3.**
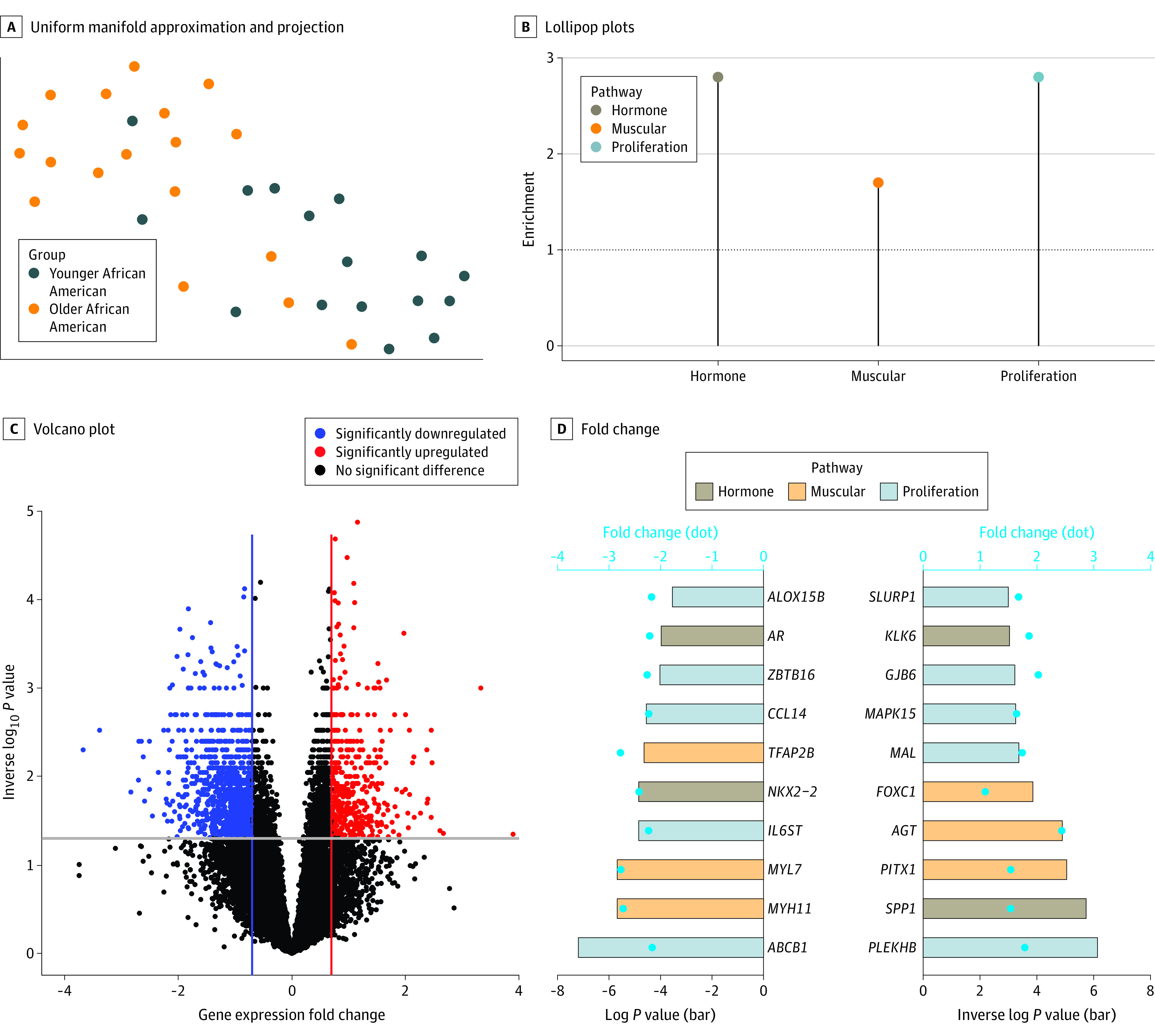
DNA Methylation, Gene Expression, and Pathway Enrichment in Younger vs Older African American Females A, The uniform manifold approximation and projection displays the distribution of triple-negative breast cancer specimens according to differentially methylated sites between younger (aged <50 years) and older (aged ≥50 years) African American females. B, Lollipop plots represent enrichment values of hormonal, muscle, and proliferation pathways. C, The volcano plot represents the gene expression fold change and −log10 of the *P* value between triple-negative breast cancer from younger and older African American females. D, The bar plot shows fold change (dots) and −log10 *P* value (bars) of genes associated with hormone, muscle, and proliferation pathways. AR indicates androgen receptor.

To investigate whether epigenetic differences detected in TNBCs of younger African American females were associated with race, we compared DNAm patterns between tumors of younger and older African American females. We identified 106 hypermethylated and 2800 hypomethylated genomic regions in younger African American females (eFigure 3A in Supplement 1). DNAm levels of these genomic regions separated younger and older African American patients in multidimensional reduction analyses (Figure 3A; eFigure 3B in Supplement 1). Genes located near genomic regions that were hypomethylated in younger African American compared with older African American patients were also enriched in hormone (OR, 2.76; 95% CI, 1.96-3.81; *P* < .001), muscle (OR, 1.65; 95% CI, 1.30-2.09; *P* < .001), and proliferation (OR, 2.76; 95% CI, 2.38-3.18; *P* < .001) pathways (Figure 3B), the same alterations detected when comparing younger African American patients with other groups.

In addition, comparative gene expression analysis revealed 405 upregulated and 914 downregulated genes in TNBC tumors of younger compared with older African American patients (Figure 3C). Several of these genes were associated with increased odds of emQTL (OR, 1.72; 95% CI, 1.53-1.93; *P* < .001) (eFigure 3C in Supplement 1). AR was also significantly downregulated (Figure 3D), suggesting that epigenetic and transcriptomic alterations in hormone, muscle, and proliferation pathways identified in younger African American patients were different from those even in older African American patients.

### Epigenetic Differences Between Younger African American and White Females

**Figure 4.**
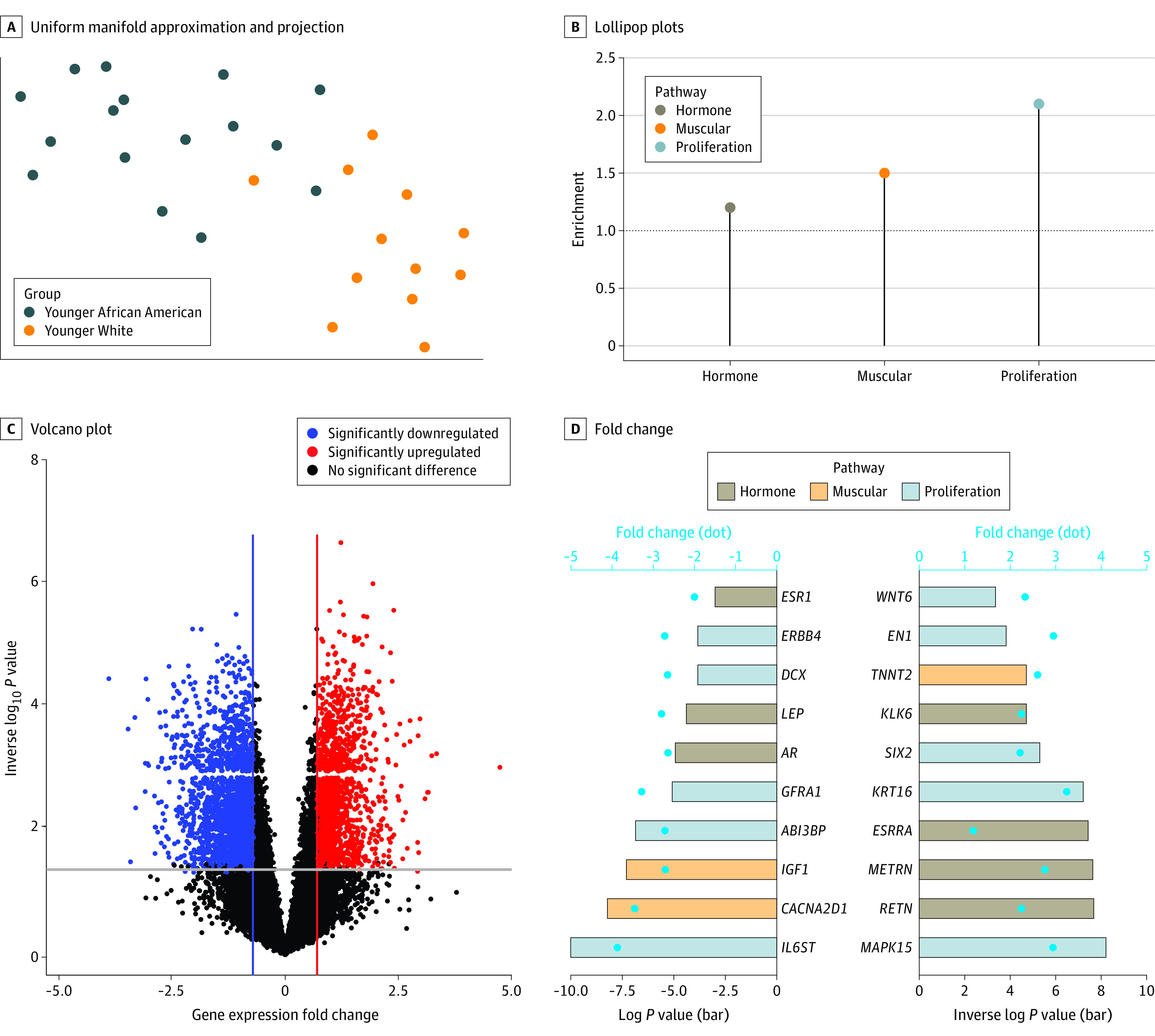
DNA Methylation, Gene Expression, and Pathway Enrichment in Younger African American Compared With Younger White Females A, The uniform manifold approximation and projection displays the distribution of triple-negative breast cancer specimens according to differentially methylated sites between younger (aged <50 years) African American and younger White females. B, Lollipop plots represent enrichment values of hormone, muscle, and proliferation pathways. C, The volcano plot represents the gene expression fold change and −log10 of the *P* value between triple-negative breast cancer from younger African American and younger White females. D, The bar plot shows fold change (dots) and −log10 *P* value (bars) of genes associated with hormone, muscle, and proliferation pathways.

Next, we examined whether alterations detected in TNBC tumors of younger African American patients were associated with age rather than race by comparing DNAm levels between younger African American and younger White patients. Analysis demonstrated 1515 differentially methylated sites (519 hypermethylated and 996 hypomethylated sites) (eFigure 4A in Supplement 1), which efficiently separated the 2 groups (Figure 4A; eFigure 4B in Supplement 1). These differentially methylated genomic regions were associated with muscle (OR, 1.50; 95% CI, 1.10-1.99; *P* = .008) and proliferation (OR, 2.14; 95% CI, 1.78-2.56; *P* < .001) but not hormone (OR, 1.23; 95% CI, 0.70-1.99; *P* = .46) pathways (Figure 4B), although a higher enrichment was seen in younger African American patients. We also observed 2137 downregulated and 1625 upregulated genes in TNBCs of younger African American compared with younger White patients (Figure 4C). Several DEGs were associated with higher odds of emQTL (OR, 1.10; 95% CI, 1.02-1.20; *P* = .02) (eFigure 4C in Supplement 1) and involved in muscle and proliferation pathways (Figure 4D). Genes involved in hormone response, such as estrogen receptor α (*ESR1*) and AR, were significantly downregulated in TNBCs of younger African American females. This observation suggests that although the association of hormones with outcomes in the TNBC epigenome was similar in younger females regardless of race, specific pathways, such as ER and AR response, were less active in TNBCs of younger African American females.

### Upstream Factors Associated With Hormone, Myoepithelial, and Proliferation Pathways in Younger African American Females

**Figure 5.**
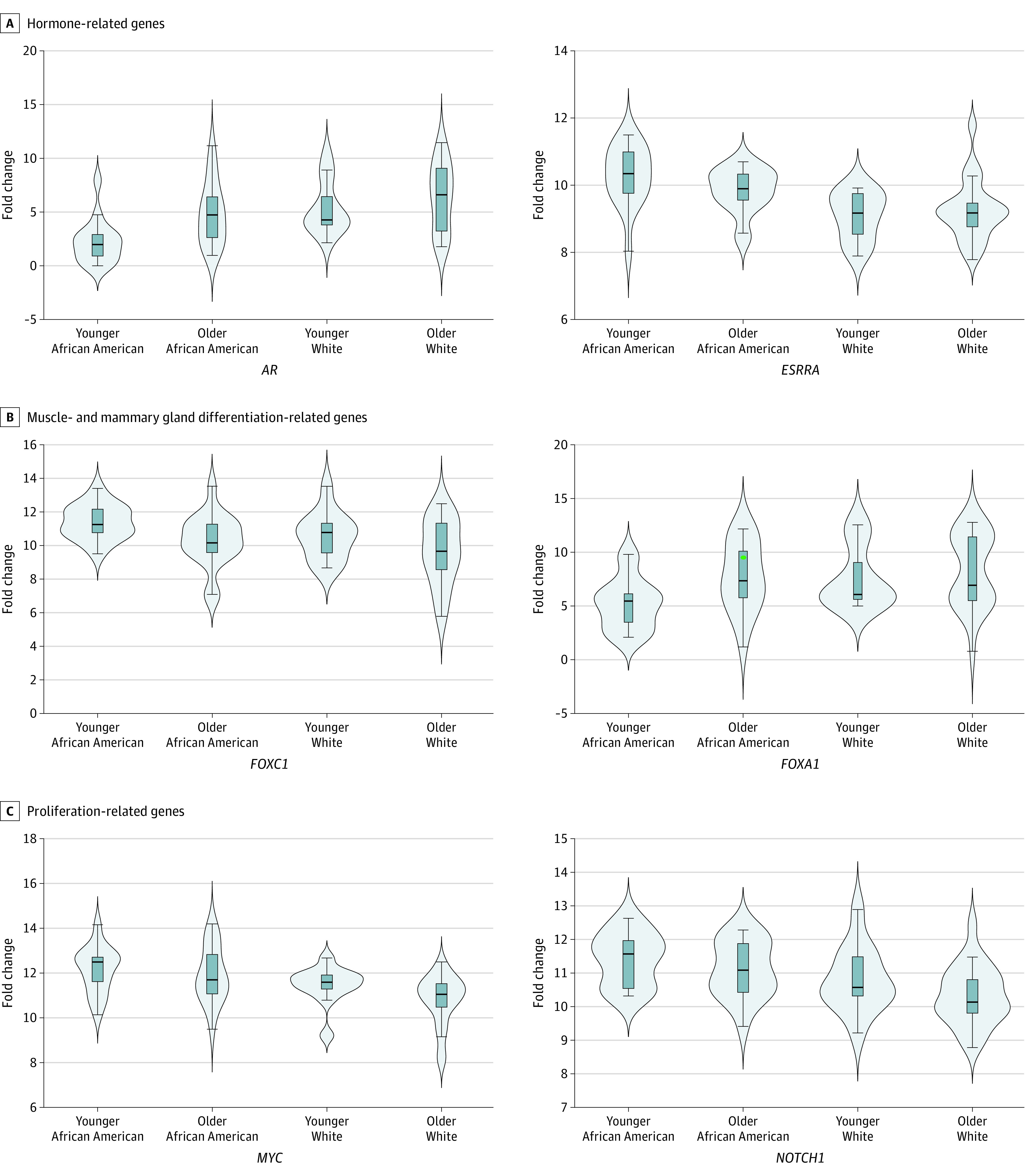
Basal-Like Phenotype–Associated Genes Differentially Expressed in Younger African American Females A, Hormone-associated genes (*AR* and *ESRRA*) are presented. B, Muscle and mammary gland differentiation–associated genes (*FOXC1* and *FOXA1*, respectively) are presented. C, Proliferation-associated genes (*MYC* and *NOTCH1*) are presented. Younger was defined as aged less than 50 years.

Based on epigenetic and transcriptomic differences of TNBCs in younger African American females, we examined associations of upstream factors with significant changes in molecular functions. As reported elsewhere,^19^ we observed a downregulation of the AR gene in hormone receptor–negative and TNBC tumors (FC = −3.40; 95% CI, −3.93 to −2.87; *P* < .001) (eFigure 5A-B in Supplement 1). Among patients with TNBC, younger African American patients had the lowest AR levels (FC = −2.93; 95% CI, −4.76 to −2.11; *P* < .001) (Figure 5A), and the expression of AR was correlated with *ESR1* expression, suggesting a common regulation of these factors (cor = 0.53; 95% CI, 0.33 to 0.68; *P* < .001) (eFigure 5C in Supplement 1). Concurrent with a lower expression level of AR, a higher expression level of *ESRRA* in hormone receptor–negative and TNBC tumors (FC = 0.86; 95% CI, 0.34 to 1.38; *P* < .001) was observed (eFigure 5D-E in Supplement 1), specifically in younger African American patients (FC = 0.86; 95% CI, 0.34-1.38; *P* = .002) (Figure 5A). *ESRRA* is an orphan nuclear receptor that has a negative correlation with *ESR1*, even in ER-positive tumors (cor = −0.29; 95% CI, −0.35 to −0.21; *P* < .001) (eFigure 5F in Supplement 1). We explored the epigenetic status of genomic regions with binding affinity for *ESRRA* and AR and observed that *ESRRA* binding sites were significantly hypomethylated in TNBCs of younger African American patients, suggesting a functional activation. In concordance, genes associated with these hypomethylated elements were significantly upregulated (eFigure 5G in Supplement 1), while genes with AR binding sites in their promoters were downregulated (eFigure 5H in Supplement 1).

Owing to its significance in regulating hormone response, AR negativity is used to define a subset of TNBCs known as quadruple-negative breast cancers (QNBCs). We observed a correlation (*r* = 0.36; *P* < .001) (eFigure 5I in Supplement 1) between gene expression profiles of TNBCs of younger African American patients and QNBC tumors of 161 patients in the Molecular Taxonomy of Breast Cancer International Consortium (METABRIC) cohort (eFigure 5I in Supplement 1).^20^ For example, while *MMP7* and *FOXC1* genes were upregulated, *MUCL1* and *FOXA1*, factors that maintain mammary cell differentiation, were downregulated in both groups. These similarities suggest that AR negativity may have been associated with the gene expression profile of TNBCs of younger African American patients, suggesting the importance of QNBCs in these patients.

### Evaluation of Myoepithelial Gene Pathways in TNBCs of Younger African American Females

Our data suggest that in addition to having a highly hormone-insensitive phenotype, TNBCs of younger African American females had a greater basal-like phenotype. *FOXC1*, a transcription factor active in myoepithelial cells,^21^ was significantly upregulated (FC = 1.33; 95% CI, 0.62 to 2.03; *P* < .001) (Figure 5B), and *FOXA1*, involved in mammary gland differentiation and modulation of ER and AR functions in breast cancer,^22^ was significantly downregulated (FC = −2.74; 95% CI, −4.24 to −1.23; *P* < .001) (Figure 5B). This pattern was also observed in patients with TNBCs compared with those with HR-positive cancers (*FOXC1*: FC = 3.17; 95% CI, 2.86 to 3.50; *P* < .001; *FOXA1*: FC = −4.73; 95% CI, −5.30 to −4.16; *P* < .001) (eFigure 6A-D in Supplement 1). *FOXC1* was negatively correlated with *FOXA1* in patients with TNBCs (cor = −0.71; 95% CI, −0.81 to −0.56; *P* < .001) (eFigure 6E in Supplement 1), highlighting their opposing role in regulating gene expression programs involved in the basal-like phenotype.^23^ Supporting this observation, we identified that all TNBCs of younger African American patients were classified as basal-like by the Prediction Analysis of Microarray 50 (PAM50) algorithm, while other groups had higher variability (eFigure 6F in Supplement 1).

### Proliferative Gene Networks Among TNBC Tumors by Group

TNBCs of younger African American patients also displayed an upregulation of genes involved in proliferation pathways, such as *MYC* (FC = 0.81; 95% CI, 0.18-1.45; *P* = .01) and *NOTCH1* (FC = 0.71; 95% CI, 0.23-1.19; *P* = .004) (Figure 5C). Additional genes involved in both pathways were significantly upregulated in these tumors, suggesting activation of these pathways in TNBCs of younger African American females (eFigure 7A-B in Supplement 1). *MYC* (FC = 0.75; 95% CI, 0.52-0.99; *P* < .001) (eFigure 8A-B in Supplement 1) and *NOTCH1* (FC = 0.97; 95% CI, 0.81-1.13; *P* < .001) (eFigure 8C-D in Supplement 1) were also upregulated in TNBC tumors compared with other breast cancer subtypes.

We validated results of this study using an external cohort of gene expression data from TNBCs of African American females generated by Purrington et al (210 patients, for a total of 279 patients in the gene expression analysis).^24^ In agreement with our core analysis, *ESR1* (FC = −0.42; 95% CI, −0.69 to −0.16; *P* = .002), *FOXA1* (FC = −0.37; 95% CI, −0.62 to −0.11; *P* = .004), and AR (FC = −0.28; 95% CI, −0.53 to −0.02; *P* = .03) were downregulated in younger compared with older African American patients in the validation analysis (eFigure 9A-C in Supplement 1). In addition, *ESRRA* (FC = 0.24; 95% CI, −0.04 to 0.53; *P* = .09), *FOXC1* (FC = 0.14; 95% CI, −0.15 to 0.42; *P* = .34), *NOTCH1* (FC = 0.14; 95% CI, −0.14 to 0.43; *P* = .33), and *MYC* (FC = 0.11; 95% CI, −0.18 to 0.40; *P* = .45) showed no significant upregulation in younger compared with older African American females (eFigure 9D-G in Supplement 1).

## Discussion

TNBC is a particularly aggressive disease, with higher rates of death than any other breast cancer subtype. Despite significant advances in breast cancer research and treatment, disparities in incidence and outcome persist between female patients of different races and ages. Specifically, younger African American females develop TNBC at more than twice the rate of older African American females and White females of any age and exhibit lower overall survival.^1^ Beyond socioeconomic and treatment variables, understanding the biological factors that may be associated with this disparity remains a major challenge. In this exploratory cross-sectional study, we identified a distinct DNAm landscape of TNBCs of younger African American patients. Furthermore, we found that the significantly hypomethylated regions identified in this patient group were enriched in genes associated with hormone, muscle, and proliferation pathways. These epigenetic differences and their associated outcomes in gene expression programs may be associated with the increased aggressivity of TNBC reported in this patient group.

Hormone pathways were consistently found to harbor epigenetic and transcriptomic alterations in younger African American females. However, we did not observe differential DNAm of hormone pathways between younger African American and younger White females, suggesting that these epigenetic mechanisms may be associated with the pathogenesis of TNBC in younger patients regardless of race. However, we identified significant differences in the expression of specific and relevant hormone pathway mediators, such as *ESR1*, AR, and *ESRRA*, suggesting a more hormone-unresponsive phenotype in TNBCs of younger African American patients, even compared with younger White patients.

Prior studies^25^ have reported that African American females have higher rates in general of QNBC, a TNBC subtype characterized by the lack of AR expression. Our data suggest that the higher incidence of QNBC may be specific to younger African American patients, demonstrated by the significant downregulation of AR in TNBC tumors of this population compared with older African American patients. Furthermore, younger African American patients exhibited a significant upregulation in *ESRRA* expression, an orphan receptor with similar binding sites to *ESR1*. Notably, *ESRRA* has been found to have a negative correlation with *ESR1* and is upregulated in TNBC in general, suggesting increased activity in patients with low estrogen signaling.^26^ Our data suggest that TNBCs of younger African American patients had even lower *ESR1* and AR expression and higher *ESRRA* expression compared with TNBCs of other patient groups, indicating a greater hormone insensitivity in this patient group.

The upregulation of *FOXC1* combined with the downregulation of *FOXA1* suggests that TNBCs of younger African American patients may have a more basal phenotype in addition to a more hormone-unresponsive phenotype. *FOXC1* is enriched in myoepithelial cells and mammary luminal progenitor cells, and therefore its upregulation and the concomitant downregulation of the mammary gland differentiation mediator *FOXA1* leads to a less differentiated phenotype.^21^ This phenomenon has been previously reported, with strong evidence indicating that *FOXC1*-high/*FOXA1*-low breast cancers tend to have basal-like phenotypes, regardless of the immunohistochemistry evaluation of ER, progesterone receptor , or HER2.^23^ Furthermore, this basal- and myoepithelial-like phenotype of TNBC of younger African American females supports a decreased hormone responsiveness given that *FOXC1* can silence *ESR1* by competing with *GATA3*.^27^

Finally, we identified epigenetic alterations associated with increased proliferation, an alteration that has been commonly described in basal-like breast tumors in general^28^ but not specifically in TNBCs of younger African American females, to our knowledge. We observed higher levels in younger African American patients of *MYC* and *NOTCH1*, 2 genes that are well known for their relevance in cancer.^29,30,31,32^ Clinically, efforts are being made to develop anti-*NOTCH* targeted therapies, primarily through the use of γ-secretase inhibitors and monoclonal antibodies against Notch receptors.^33^ Younger African American patients with TNBC showing disproportionate enhancement of *NOTCH1* could potentially benefit from these novel therapies.

### Limitations

This study has several limitations. The main limitation is that paired DNAm and gene expression data from patients with TNBC and well-annotated data, including race and age, were unavailable, resulting in our small sample size. This also precluded the ability to perform meaningful adjusted analyses. Additionally, our study involved the stratification of patients based on self-reported race. In the absence of ancestry tests to confirm race genetically, there is a risk that race groups in our study were misrepresented.

## Conclusions

In this exploratory cross-sectional study, we found that the epigenetic landscape of TNBC tumors of younger African American patients was epigenetically distinct, providing important insight into the potential mechanisms contributing to the aggressive behavior of this cancer within this patient group. Our findings have the potential to improve the clinical treatment of these individuals by unveiling possible targets for novel therapeutics and underscoring the significance of accounting for race- and age-associated epigenetic variations in TNBC. Future research should focus on discovering genomic regions contributing to these epigenetic discrepancies and their functional implications in associated changes in the behavior of TNBC in younger African American females.

## Notes

Supplement 1. **eFigure 1.** DNA Methylation Differences Between African American and White Females **eFigure 2.** Gene Expression Differences in Younger African American Females vs Other Groups **eFigure 3.** DNA Methylation and Gene Expression Differences Between Younger vs Older African American Females **eFigure 4.** DNA Methylation and Gene Expression Differences Between Younger African American vs Younger White Females **eFigure 5.** Differential Expression of *AR* and *ESRRA* by Breast Cancer Subtype, Correlation with *ESR1*, and Associated Gene Expression **eFigure 6.** *FOXC1* and *FOXA1* Expression Levels by Breast Cancer Subtype **eFigure 7.** Aberrantly Active Pathways on Triple-Negative Breast Cancer Tumors of Younger African American Females **eFigure 8.** Analysis of Proliferation Genes in Triple-Negative Breast Cancer **eFigure 9.** Key Genes Associated With Hormone, Muscle, and Proliferation Pathways in Validation Cohort

Supplement 2.
Data Sharing Statement
