# Supplementary material for: Epigenetic Profiles of Triple-Negative Breast Cancers of African American and White Females
Source: JAMA Netw Open. 2023 Oct 5;6(10):e2335821. doi: 10.1001/jamanetworkopen.2023.35821 (PMC10556970; doi:10.1001/jamanetworkopen.2023.35821)
Supplement: Supplement 1. — eFigure 1. DNA Methylation Differences Between African American and White Females eFigure 2. Gene Expression Differences in Younger African American Females vs Other Groups eFigure 3. DNA Methylation and Gene Expression Differences Between Younger vs Older African American Females eFigure 4. DNA Methylation and Gene Expression Differences Between Younger African American vs Younger White Females eFigure 5. Differential Expression of AR and ESRRA by Breast Cancer Subtype, Correlation with ESR1, and Associated Gene Expression eFigure 6. FOXC1 and FOXA1 Expression Levels by Breast Cancer Subtype eFigure 7. Aberrantly Active Pathways on Triple-Negative Breast Cancer Tumors of Younger African American Females eFigure 8. Analysis of Proliferation Genes in Triple-Negative Breast Cancer eFigure 9. Key Genes Associated With Hormone, Muscle, and Proliferation Pathways in Validation Cohort [file jamanetwopen-e2335821-s001.pdf]

Supplemental Online Content

Ensenyat-Mendez M, Solivellas-Pieras M, Llinàs-Arias P, et al. Epigenetic profiles of triple-negative breast cancers of African American and White females. *JAMA Netw Open*. 2023;6(10):e2335821. doi:10.1001/jamanetworkopen.2023.35821

eFigure 1. DNA Methylation Differences Between African American and White Females

eFigure 2. Gene Expression Differences in Younger African American Females vs Other Groups

eFigure 3. DNA Methylation and Gene Expression Differences Between Younger vs Older African American Females

eFigure 4. DNA Methylation and Gene Expression Differences Between Younger African American vs Younger White Females

eFigure 5. Differential Expression of *AR* and *ESRRA* by Breast Cancer Subtype, Correlation with *ESR1*, and Associated Gene Expression

eFigure 6. *FOXC1* and *FOXA1* Expression Levels by Breast Cancer Subtype

eFigure 7. Aberrantly Active Pathways on Triple-Negative Breast Cancer Tumors of Younger African American Females

eFigure 8. Analysis of Proliferation Genes in Triple-Negative Breast Cancer

eFigure 9. Key Genes Associated With Hormone, Muscle, and Proliferation Pathways in Validation Cohort

This supplemental material has been provided by the authors to give readers additional information about their work.

© 2023 Ensenyat-Mendez M et al. *JAMA Network Open.*

Supplementary eFigure Legends

eFigure 1. DNA Methylation Differences Between African American and White Females. (A) Volcano plot displaying the fold change (x-axis) and the -log10 p-value of DNAm differences between TNBC from African American and White Females. **(B)** Hierarchical clustering using all DMS between TNBC tumors of African American and White patients. Correlation plots displaying the DNAm pattern in TNBC of Younger African American and other patients when using: (**C**) 5,000, (**D**), 2,000, (**E**), 1,000, (**F**), 500, (**G**), 200, and (**H**), 100 most variable regions.

eFigure 2. Gene Expression Differences in Younger African American Females vs Other Groups. (A) Volcano plot displaying the fold change (x-axis) and the -log10 p-value of DNAm differences between TNBC from Younger African Americans and those tumors affecting the rest of the race and age categories. **(B)** Hierarchical clustering using all DMS between TNBC tumors affecting Younger African Americans and the rest. (**C**) Venn diagram representing the enrichment of the differentially expressed genes (DEG) in TNBC from Younger African Americans compared with the rest controlled by emQTLs in the comparison. An illustrative example of MFSD6L, a gene regulated by emQTL showing the DMS overlapping with the reported emQTLs. (**D-E**) Gene Enrichment Set Analysis (GSEA) plot displaying the (**D**) negative enrichment of genes related to hormone response and (**E**) a positive enrichment of genes related to the cell cycle.

eFigure 3. DNA Methylation and Gene Expression Differences between Younger vs Older African American Females. (A) Volcano plot displaying the fold change (x-axis) and the -log10 p-value of DNAm differences between TNBC from Younger African Americans and those tumors affecting Older African Americans. **(B)** Hierarchical clustering using all DMS between TNBC tumors affecting Younger African Americans and Older African Americans. (**C**) Venn diagram representing the enrichment of the differentially expressed genes (DEG) in TNBC from Younger African Americans compared with Older African Americans controlled by emQTLs in the comparison. An illustrative example of PKP3, a gene regulated by emQTL showing the DMS overlapping with the reported emQTLs.

© 2023 Ensenyat-Mendez M et al. *JAMA Network Open.*

eFigure 4. DNA Methylation and Gene Expression Differences Between Younger African Americans vs Younger White Females. (A) Volcano plot displaying the fold change (x-axis) and the -log10 p-value of DNAm differences between TNBC from Younger African Americans and Younger White Female. **(B)** Hierarchical clustering using all DMS between TNBC tumors affecting Younger African Americans and Younger White Female. (**C**) Venn diagram representing the enrichment of the differentially expressed genes (DEG) in TNBC from Younger African Americans compared with Younger White Female controlled by emQTLs in the comparison. An illustrative example of S100A16, a gene regulated by emQTL showing the DMS overlapping with the reported emQTLs.

eFigure 5. Differential Expression of AR and ESRRA by Breast Cancer Subtype, Correlation with ESR1, and Associated Gene Expression. (A, B) Violin plots representing the expression of AR in **(A)** different breast cancer subtypes and **(B)** hormone receptor (HR)-positive vs. HR-negative breast cancer tumors. **(C)** Correlation between the expression of AR and ESR1 in TNBC tumors. **(D, E)** Violin plot representing the expression of ESRRA in **(D)** breast cancer subtypes and **(E)** HR-positive and HR-negative breast cancer tumors. **(F)** Correlation between the expression of ESRRA and ESR1 in breast tumors. Volcano plot representing the fold change (x-axis) and the -log10 p-value (y-axis) of genes differentially expressed on TNBC of Younger African Americans when compared to TNBC affecting the rest of the patients associated with **(G)** ESRRA binding sites and **(H)** AR binding sites. **(I)** Scatter plot representing fold change of all genes in QNBC compared to non-QNBC (x-axis) and fold change of all genes in TNBC affecting Younger African Americans patients compared to those affecting the rest of the race and age categories.

eFigure 6. FOXC1 and FOXA1 Expression Levels by Breast Cancer Subtype. (A, B) Violin plot representing the gene expression of FOXC1 in **(A)** breast cancer subtypes and **(B)** HR-positive and HR-negative breast cancer tumors. **(C, D)** Violin plot representing the gene expression of FOXA1 in **(C)** the breast cancer subtypes and **(D)** HR-positive and HR-negative breast cancer tumors. **(E)** Correlation plot between FOXC1 and FOXA1 expression levels in TNBC. **(F)** Stacked plots displaying the percentage of Basal, Luminal-A, Luminal-

© 2023 Ensenyat-Mendez M et al. *JAMA Network Open.*

B, HER2-enriched, and Normal-like tumors in Younger and Older African American and White patients with TNBC.

eFigure 7. Aberrantly Active Pathways on Triple-Negative Breast Cancer Tumors of Younger African American Females. Representation of **(A)** MYC-related cell cycle pathway and **(B)** Notch signaling pathway. Additional differentially upregulated (red boxes) and downregulated (green boxes) are included in each pathway.

eFigure 8. Analysis of Proliferation Genes in Triple-Negative Breast Cancer. (A, B) Violin plot representing the gene expression of MYC in **(A)** breast cancer subtypes and **(B)** HR-positive and HR-negative breast cancer tumors. **(C, D)** Violin plot representing the gene expression of NOTCH1 in **(C)** breast cancer subtypes and **(D)** HR-positive and HR-negative breast cancer tumors.

eFigure 9. Key Genes Associated with Hormone, Muscle, and Proliferation Pathways in Validation Cohort. Violin plots representing the expression levels between Younger and Older African American Females of **(A)** ESR1, **(B)** FOXA1, **(C)** AR, **(D)** ESRRA, **(E)** FOXC1, **(F)** NOTCH1, and **(G)** MYC.

© 2023 Ensenyat-Mendez M et al. *JAMA Network Open.*

eTable 1: Patient demographic and tumor characteristics

Variable	Classes	Younger African American (n=16)	Older African American (n=18)	Younger White (n=12)	Older White (n=23)	p-value	test
Age - yr (mean ± sd)		45.8 ± 4.12	64.9 ± 9.4	44.2 ± 6.4	61.4 ± 8.3	< 0.001	Kruskal-Wallis
Nodal status - no (%)	pN0	9 (56.3)	12 (66.7)	6 (50)	15 (65.2)	0.91	Fisher
	pN1	5 (31.3)	3 (16.7)	3 (25)	5 (21.7)		
	pN2-pN3	2 (12.5)	3 (16.7)	3 (25)	3 (13)		
Stage - no (%)	Stage I	3 (18.8)	2 (11.1)	2 (16.7)	6 (27.3)	0.82	Fisher Exact Test
	Stage II	11 (68.8)	13 (72.2)	7 (58.3)	14 (63.6)		
	Stage III	2 (12.5)	3 (16.7)	3 (25)	2 (9.1)		
Histology - no (%)	Infiltrating Carcinoma NOS	0 (0)	1 (5.6)	0 (0)	0 (0)	0.23	Fisher Exact Test
	Infiltrating Ductal Carcinoma	14 (87.5)	15 (83.3)	9 (75)	18 (81.8)		
	Infiltrating Lobular Carcinoma	1 (6.3)	1 (5.6)	0 (0)	0 (0)		
	Medullary Carcinoma	0 (0)	1 (5.6)	2 (16.7)	0 (0)		
	Metaplastic Carcinoma	0 (0)	0 (0)	1 (8.3)	3 (13.6)		
	Other	1 (6.3)	0 (0)	0 (0)	1 (4.5)		
Tumor site - no (%)	Left - undetermined	4 (25)	2 (11.1)	0 (0)	5 (21.7)	0.69	Fisher Exact Test
	Left LIQ	0 (0)	0 (0)	1 (8.3)	1 (4.3)		
	Left LOQ	1 (6.3)	2 (11.1)	1 (8.3)	0 (0)		
	Left UIQ	0 (0)	1 (5.6)	1 (8.3)	1 (4.3)		
	Left UOQ	3 (18.8)	5 (27.8)	3 (25)	5 (21.7)		
	Right - undetermined	1 (6.3)	3 (16.7)	2 (16.7)	2 (8.7)		
	Right LIQ	0 (0)	0 (0)	0 (0)	0 (0)		
	Right LOQ	2 (12.5)	0 (0)	0 (0)	0 (0)		
	Right UIQ	0 (0)	2 (11.1)	1 (8.3)	1 (4.3)		
	Right UOQ	5 (31.3)	3 (16.7)	3 (25)	8 (34.8)		

© 2023 Ensenyat-Mendez M et al. *JAMA Network Open.*

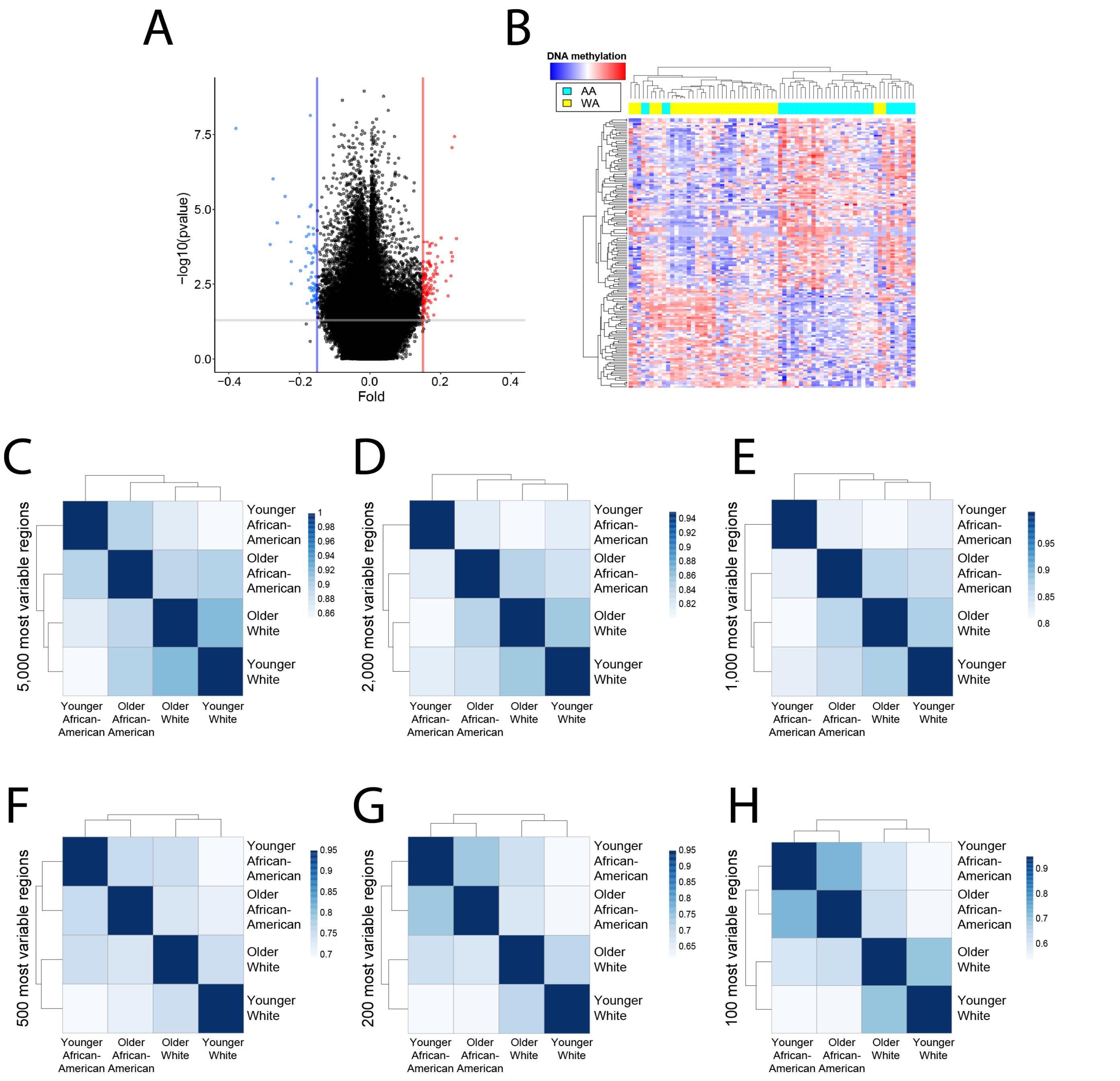

eFigure 1

© 2023 Ensenyat-Mendez M et al. *JAMA Network Open.*

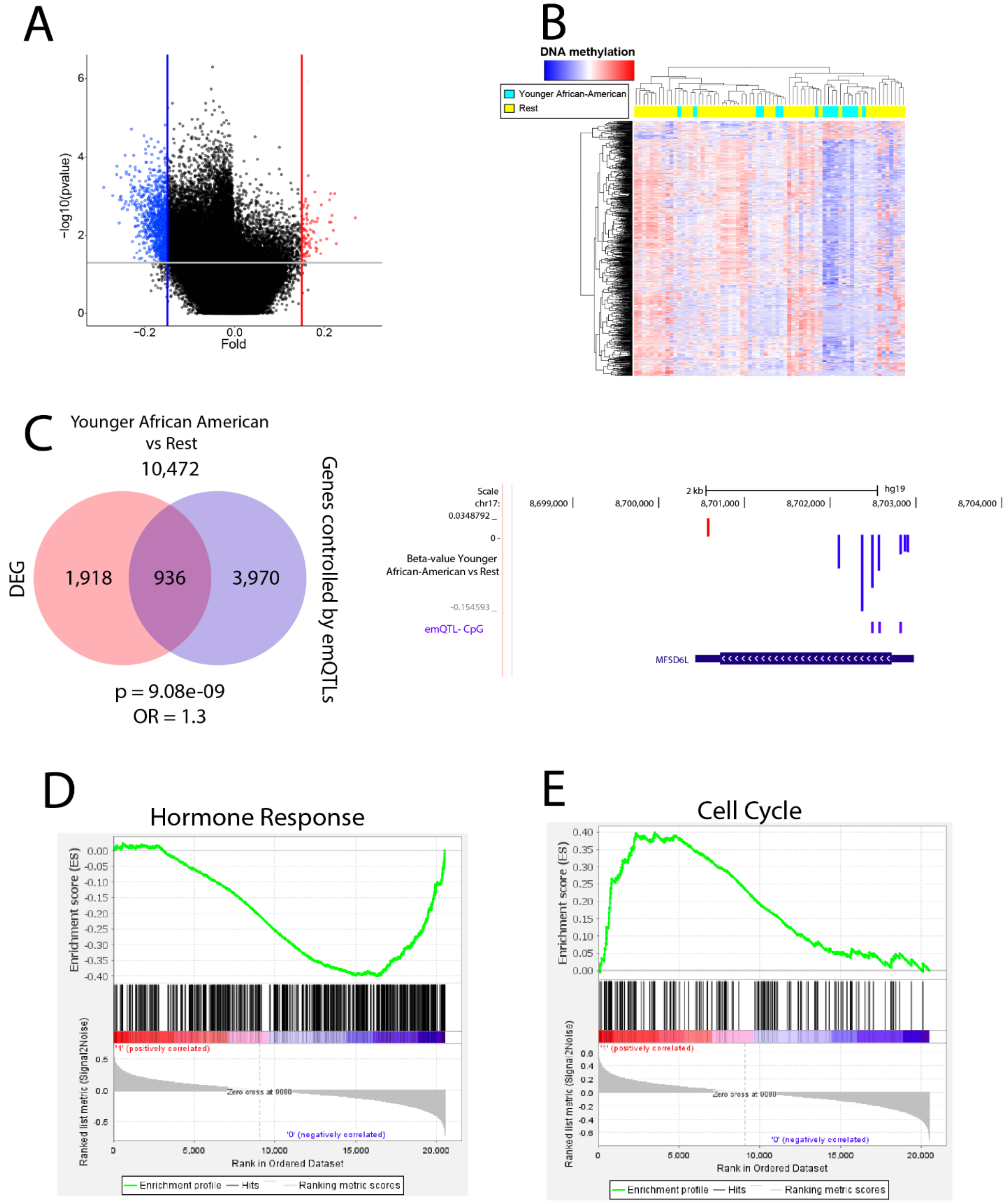

eFigure 2

© 2023 Ensenyat-Mendez M et al. *JAMA Network Open.*

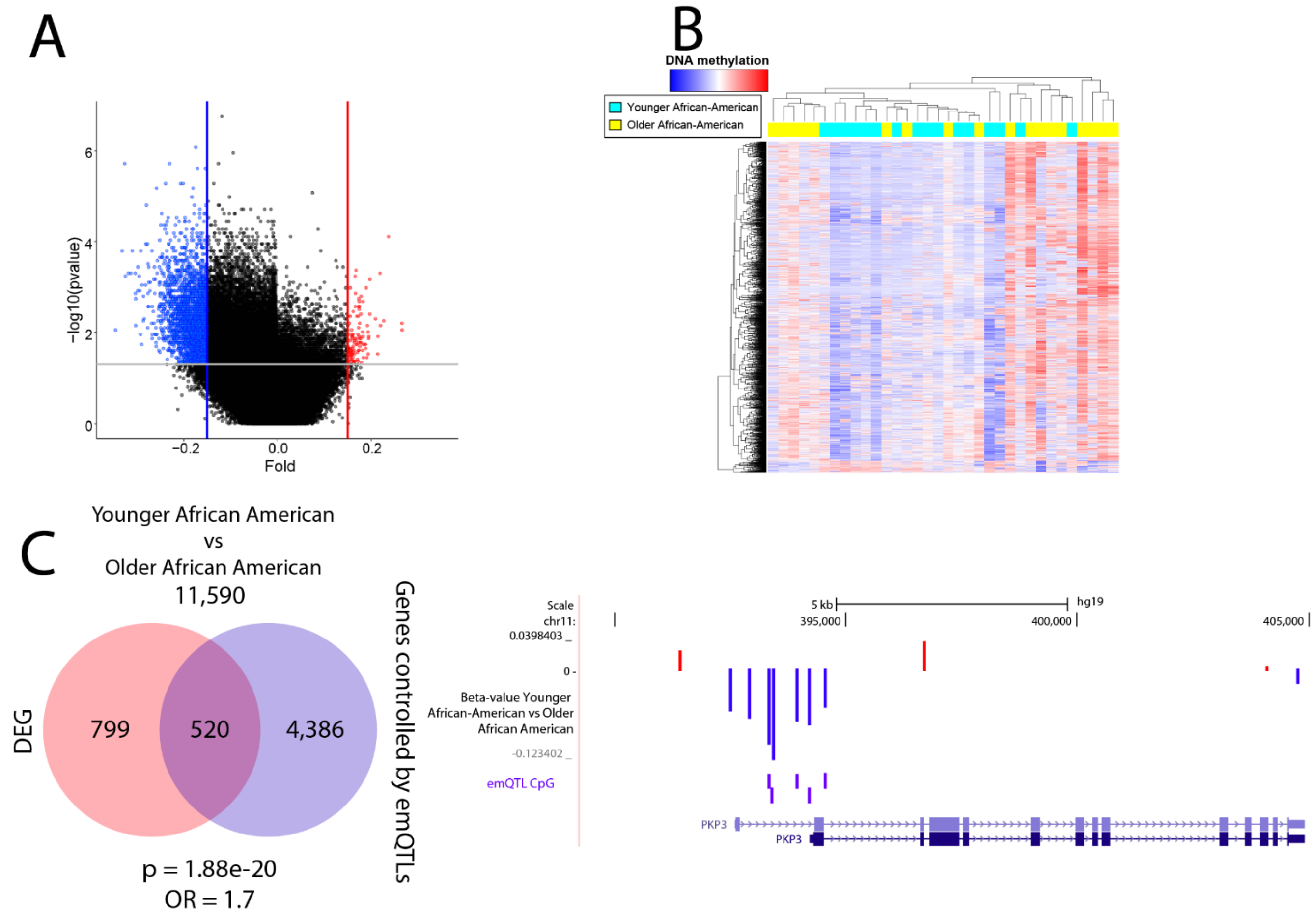

eFigure 3

© 2023 Ensenyat-Mendez M et al. *JAMA Network Open.*

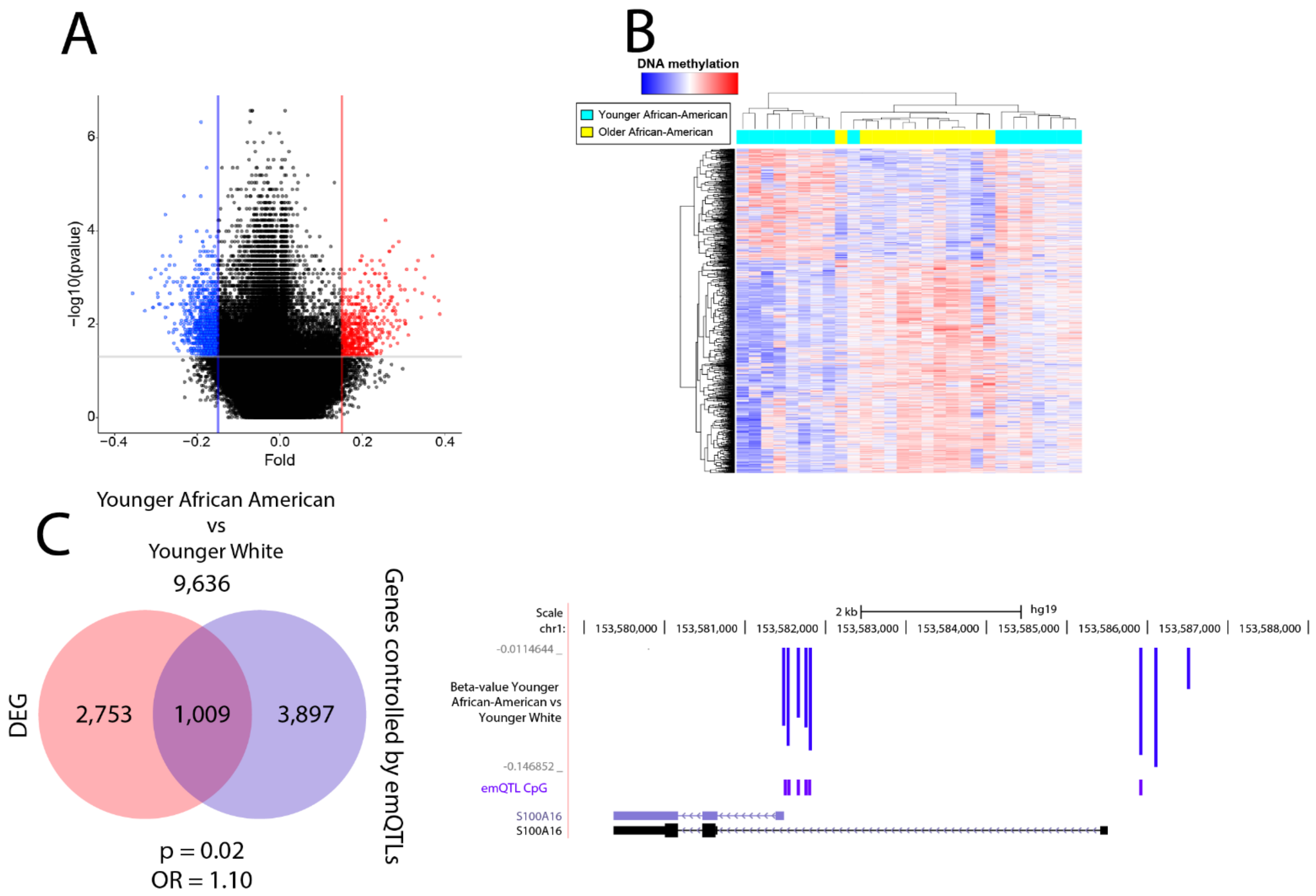

eFigure 4

© 2023 Ensenyat-Mendez M et al. *JAMA Network Open.*

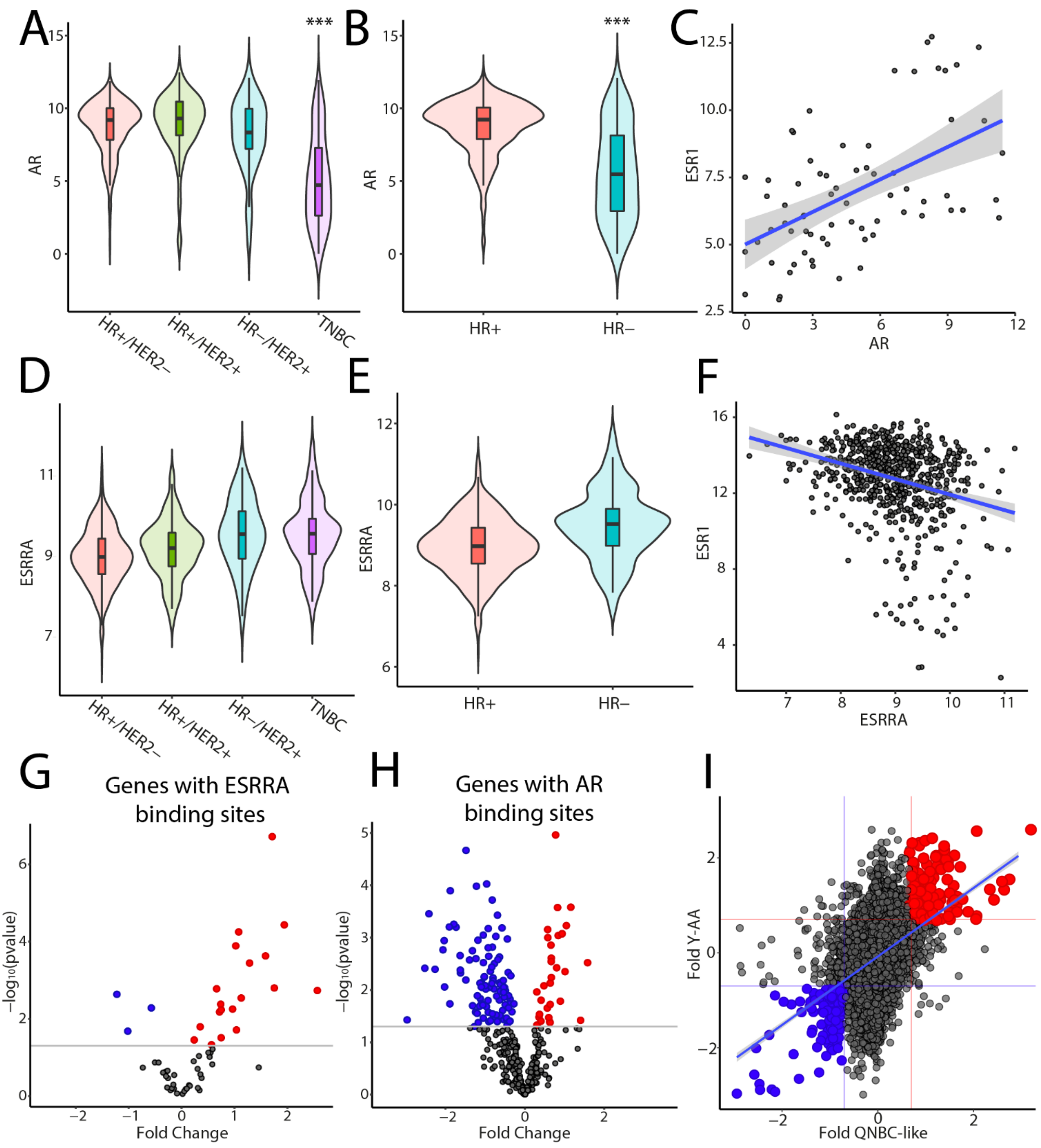

eFigure 5

© 2023 Ensenyat-Mendez M et al. *JAMA Network Open.*

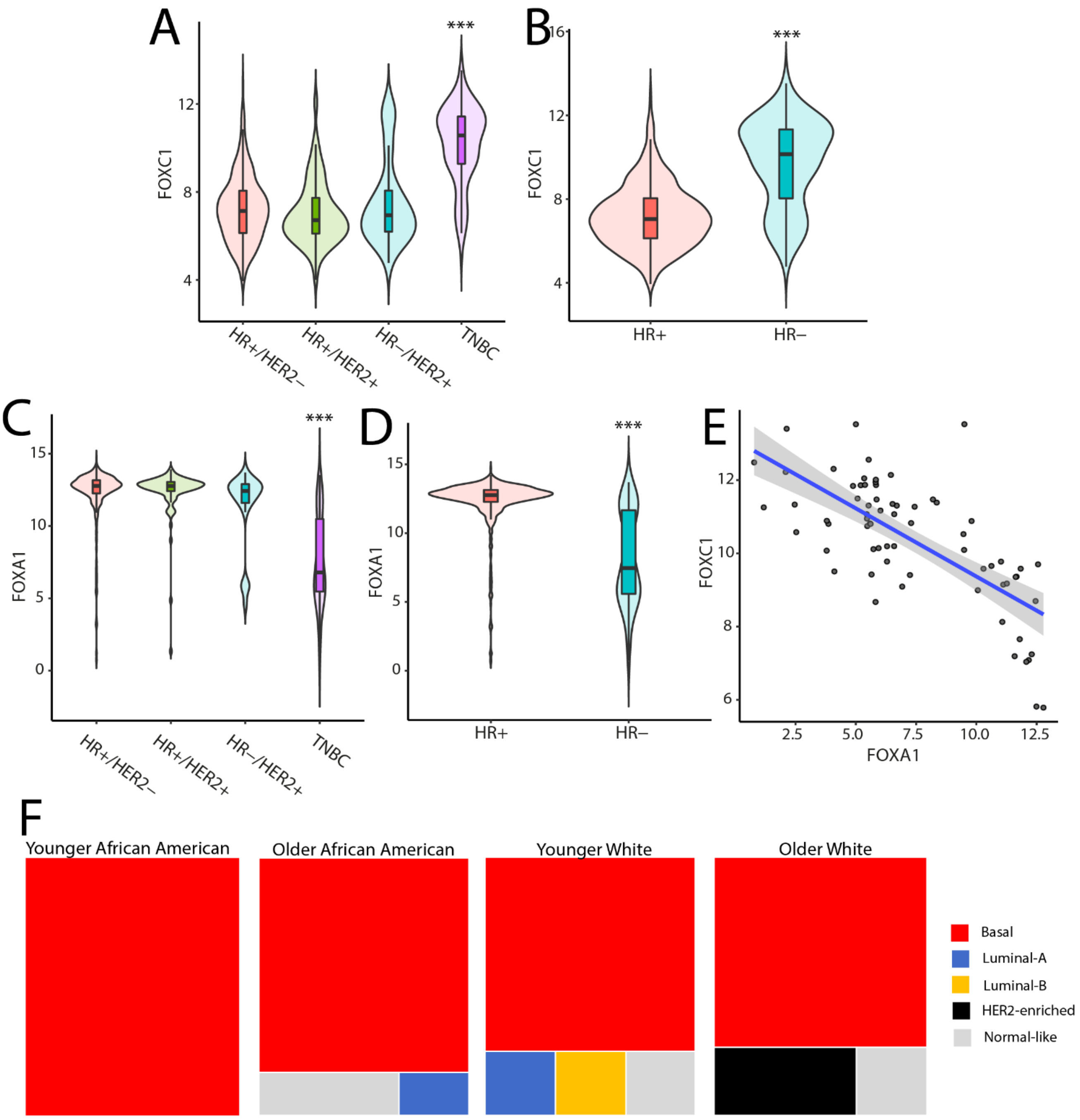

eFigure 6

© 2023 Ensenyat-Mendez M et al. *JAMA Network Open.*

A

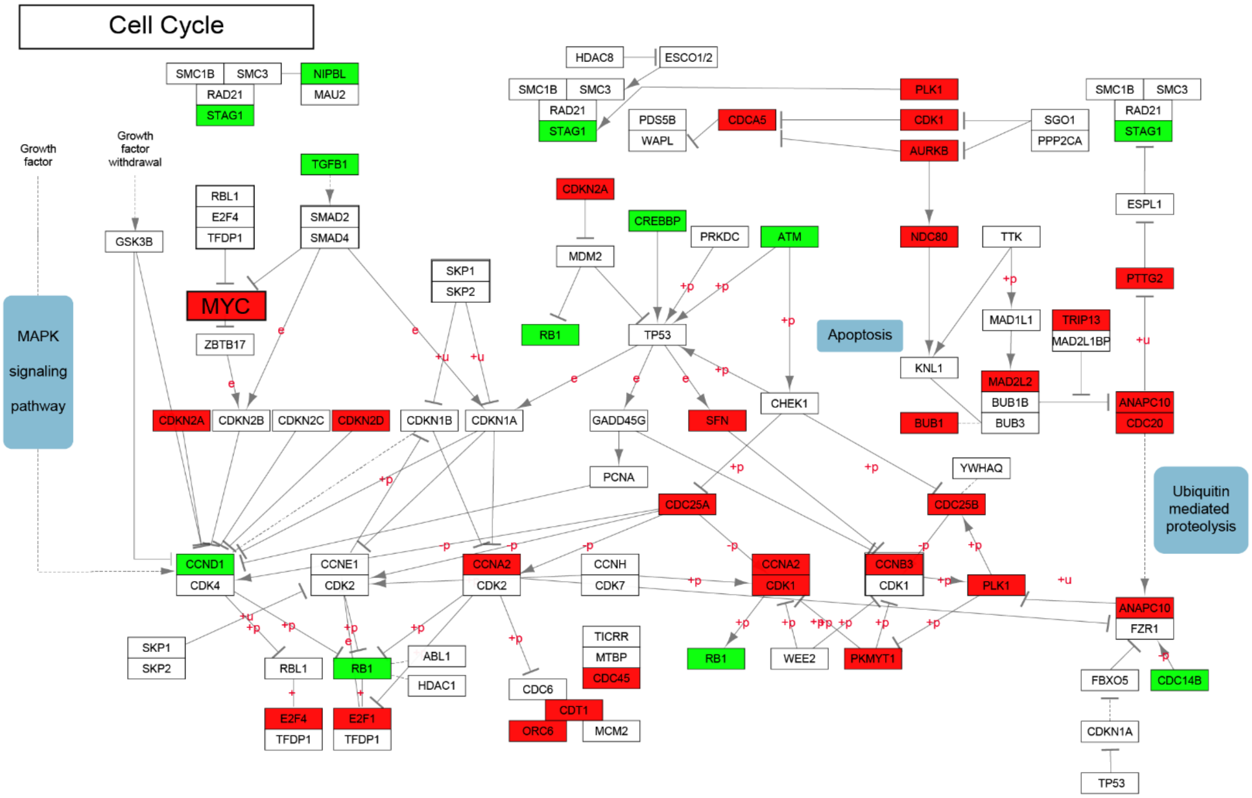

B

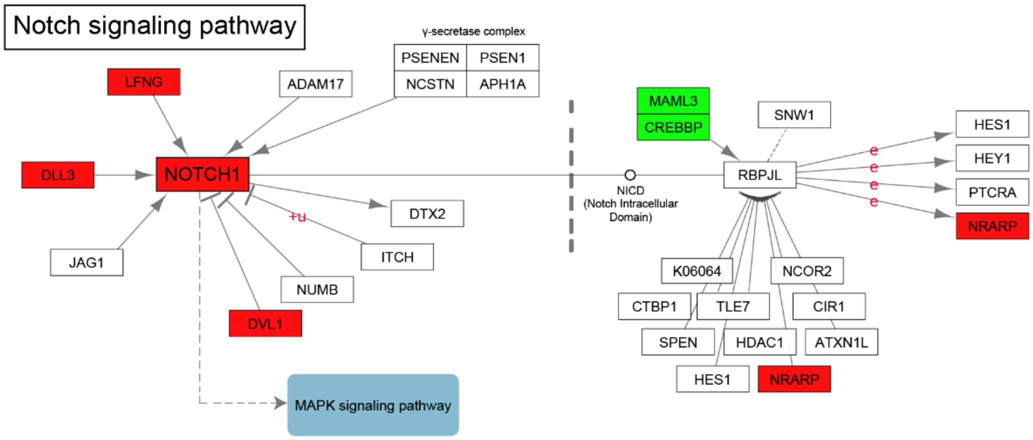

eFigure 7

© 2023 Ensenyat-Mendez M et al. *JAMA Network Open.*

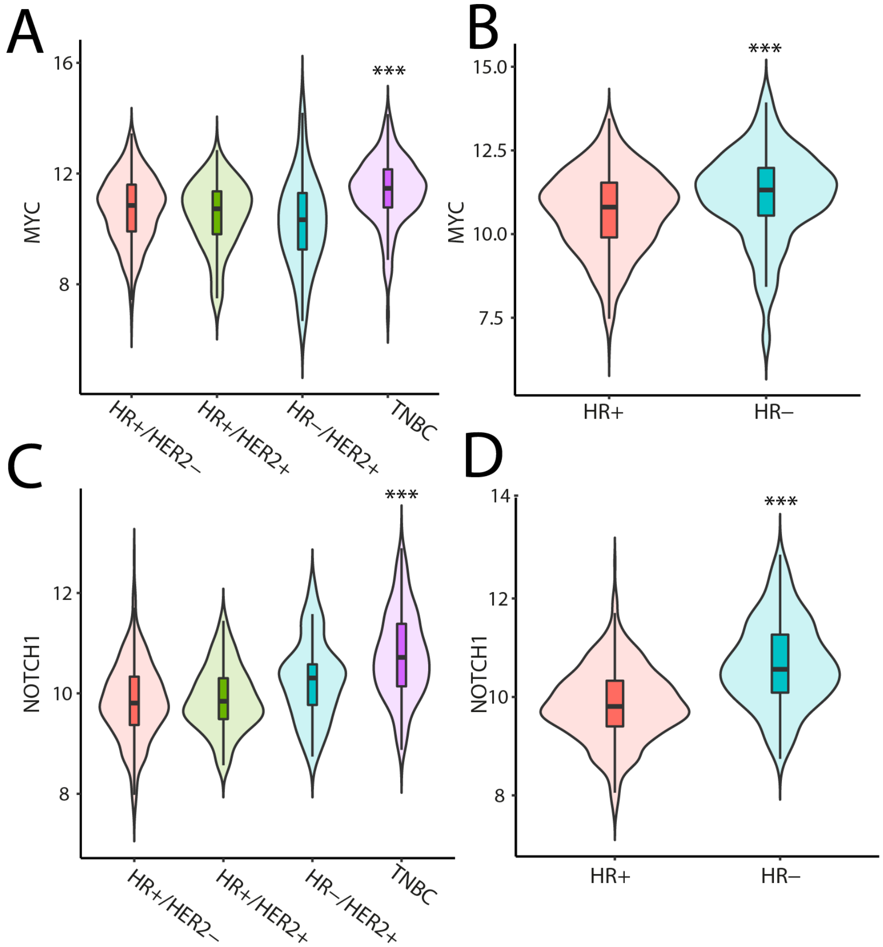

eFigure 8

© 2023 Ensenyat-Mendez M et al. *JAMA Network Open.*

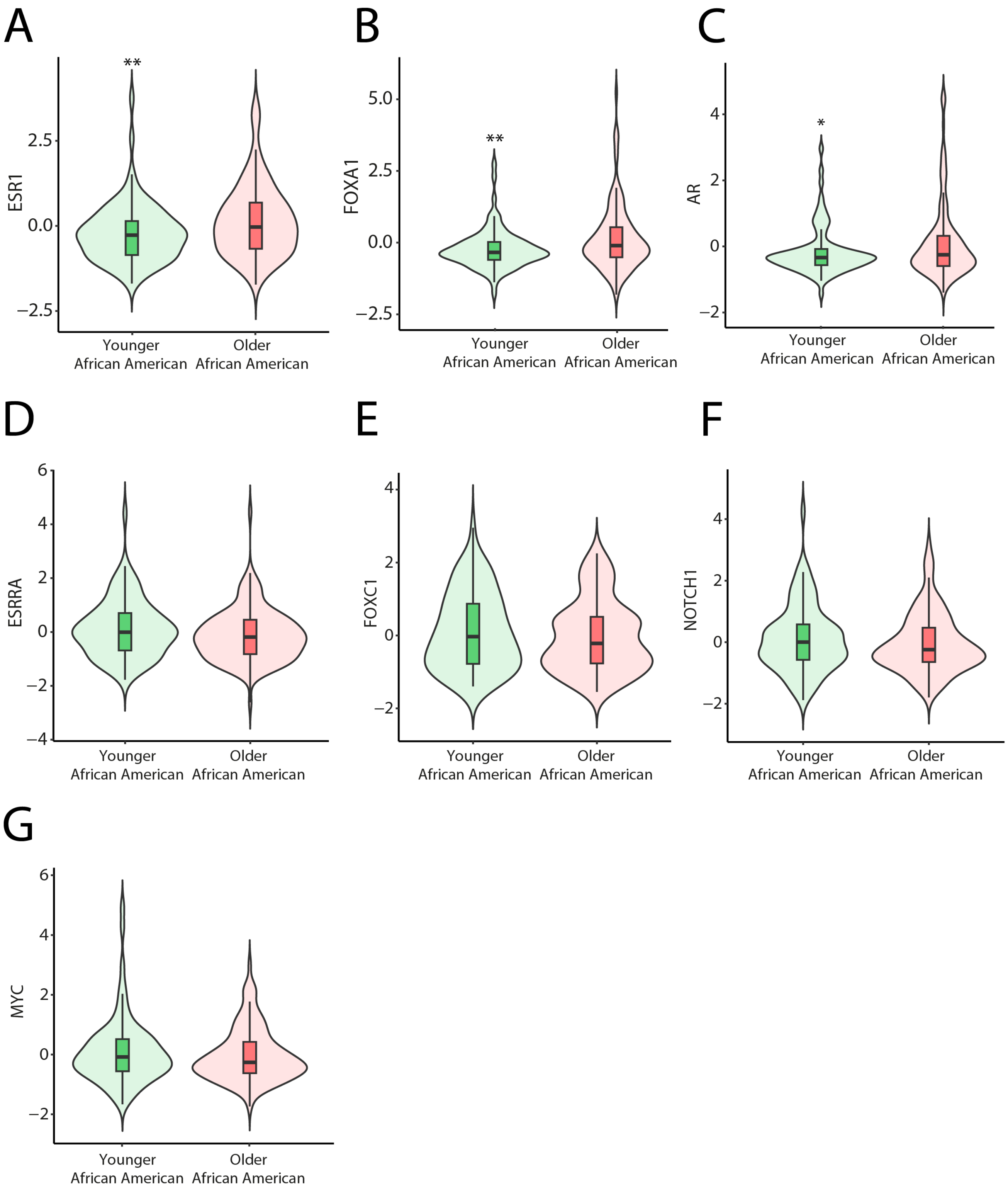

eFigure 9

© 2023 Ensenyat-Mendez M et al. *JAMA Network Open.*
