# Supplementary material for: Epigenetic Profiles of Triple-Negative Breast Cancers of African American and White Females
Source: JAMA Netw Open. 2023 Oct 5;6(10):e2335821. doi: 10.1001/jamanetworkopen.2023.35821 (PMC10556970; doi:10.1001/jamanetworkopen.2023.35821)
Supplement: Supplement 2. — Data Sharing Statement [file jamanetwopen-e2335821-s002.pdf]

Data Sharing Statement

Ensenyat-Mendez. Epigenetic Profiles of Triple-Negative Breast Cancers of African American and White Females. *JAMA Netw Open*. Published online October 5, 2023. doi:10.1001/jamanetworkopen.2023.35821

Data
Data available: No

Additional Information
Explanation for why data not available: The results in our study are in whole or part based upon data generated by the TCGA Research Network: https://www.cancer.gov/tcga and is publicly available.
